# Biosurfactant Production by Lactic Acid Bacterium *Pediococcus dextrinicus* SHU1593 Grown on Different Carbon Sources: Strain Screening Followed by Product Characterization

**DOI:** 10.1038/s41598-019-41589-0

**Published:** 2019-03-27

**Authors:** Abouzar Ghasemi, Marzieh Moosavi-Nasab, Payam Setoodeh, Gholamreza Mesbahi, Gholamhossein Yousefi

**Affiliations:** 1 Department of Food Science and Technology, School of Agriculture, Shiraz University, Shiraz, Iran; 2 Seafood Processing Research Group, School of Agriculture, Shiraz University, Shiraz, Iran; 3 Department of Chemical Engineering, School of Chemical and Petroleum Engineering, Shiraz University, Shiraz, Iran; 4 Department of Pharmaceutics, School of Pharmacy, Shiraz University of Medical Sciences, Shiraz, Iran

**Keywords:** Metabolic engineering, Applied microbiology

## Abstract

The present study focused on producing and characterizing a type of biosurfactant (BS) derived from lactic acid bacteria (LAB) and its potential applications in pharmaceutical and food industries due to the preference of employing nonpathogenic organisms in bioprocesses. To this aim, several screening approaches were applied to identify an efficient BS-producing strain from a set of LAB, and *Pediococcus dextrinicus* SHU1593 was selected as the most operative one. The BS produced by *P. dextrinicus* was isolated and structurally characterized as a lipoprotein with an approximately equal ratio of lipids (~52% (w/w)) and proteins (47% (w/w)). It reduced the surface tension (ST) of phosphate-buffered saline (PBS) from 72.80 ± 0.10 to 39.01 ± 0.32 mN/m. The results also indicated the potential of developing low-cost strategies aimed at the production of efficient LAB-derived BSs which are structurally and quantitatively similar to the ones obtained from conventional media. Finally, given the physical and functional characterization (i.e. critical micelle concentration (CMC), emulsification index (%E_24_), stability, as well as antimicrobial and anti-adhesive activities) of the BS produced in the present study, it can be introduced as a promising candidate to be employed in plenty of areas in pharmaceutical and food industries.

## Introduction

Surfactants are amphiphilic molecules consisting of both hydrophilic (polar) and hydrophobic (nonpolar) moieties. Accumulating at the interface, these chemicals can decrease the surface tension (ST) and the interfacial tension between aqueous and other immiscible solutions^1^. Surfactants are synthesized chemically from petrochemical and oleochemical resources^2^. Nowadays, due to global environmental awareness, the utilization of biological surface-active agents produced from living organisms has attracted scholarly attention^3^. Biosurfactants (BSs) are natural surface-active compounds synthesized by a broad range of microorganisms. They have acquired increased attention due to their diverse applicability, biodegradability, low toxicity, effectiveness at extreme conditions of pH and temperature, and ability to be produced from inexpensive substrates^2,4^.

Two important factors should be considered in the production of BSs, particularly those used for pharmaceutical and food industries. First, BS production should be done by nonpathogenic and safe organisms in order to avoid the problems stemming from pathogenicity. This is despite the fact that the most BS-producing microorganisms are pathogenic, and hence their exploitation in large-scale industrial processes would be excessively troublesome^5,6^. Second, BS production should involve using cheap substrates in order to decrease the overall costs of fermentative processes. In other words, an effective BS-productive process develops an economic system that makes use of low-cost materials while it provides a high-product yield^7^.

Lactic acid bacteria (LAB), which are generally regarded as safe microorganisms (GRAS, the United States Food and Drug Administration), are recognized for their use in medicine and food industries. Due to their nonpathogenicity, LAB have been the focus of scientific attention in the process of BS production. Various studies have reported that different species of LAB are able to biosynthesize surface-active molecules^5,8–10^. The BSs generated by LAB exhibit the ability to possess anti-adhesive activity against some pathogenic microorganisms and to inhibit pathogenic bacteria and fungi^9,11,12^. However, there is inadequate information about the chemical composition of BSs derived from LAB, mainly due to their complexity. Studies on the chemical composition of these BSs suggest that they comprise of different components depending on the type of strain: glycolipids^5,13^, glycoproteins^14^ or multi-component mixtures of proteins and polysaccharides associated with phosphate groups^10^. In food industries, BSs can be utilized for the stabilization of aerated systems, the formation of emulsions, the modification of rheological properties, and the improvement of the texture and consistency of products including fat and starch. BSs may also have antimicrobial and anti-adhesive properties; therefore, they can control the growth of pathogenic microorganisms on surfaces which are in contact with food materials^15^.

In the current study, several screening methods were applied to identify and select an efficient BS-producing strain from a set of numerous LAB. The production of BSs by the selected strain (*Pediococcus dextrinicus* SHU1593) using low-cost alternative media, molasses and date syrup, was examined and compared with the conventional synthetic medium. Afterwards, the characterization of the BS was evaluated based on the determination of minimum ST, critical micelle concentration (CMC), emulsification index (%E_24_) and stability to different factors such as pH and temperature. Besides, its partial functional characterization was established by determining the antimicrobial and anti-adhesive activities. Finally, the structural properties of the synthesized BSs were studied.

## Methods

### Strains and Culture Conditions

Eighteen LAB strains were obtained from the Culture Collection of Shiraz University, Iran (See Table 1). The strains were stored at −80 °C in MRS containing 15% (v/v) glycerol solution so that they can be utilized. MRS medium was prepared including 20 g/l D-glucose, 10 g/l peptone, 8 g/l meat extract, 4 g/l yeast extract, 2 g/l di-potassium hydrogen phosphate, 2 g/l di-ammonium hydrogen citrate, 5 g/l sodium acetate, 0.2 g/l magnesium sulphate, 0.05 g/l manganese sulphate, and 1 g/l Tween-80. The pH of the medium was adjusted to 7.0 and sterilized by autoclaving at 121 °C for 15 min. Whenever required, the stocks were streaked on MRS agar plates and incubated overnight at 37 °C for further cultivations. The agar plates were stored at 4 °C for no longer than 2 weeks.

**Table 1:** Types of strains used in this study.

Strain	Collection No.
*Lactobacillus salivarius*	SHU1388
*Lactobacillus acidophilus*	SHU1365
*Lactobacillus casei*	SHU1400
*Lactobacillus plantarum*	SHU1391
*Lactobacillus plantarum*	SHU1394
*Lactobacillus casei*	SHU1396
*Lactobacillus reuteri*	SHU1965
*Lactobacillus fermentum*	SHU6343
*Leuconostoc mesenteroides*	SHU1368
*Lactobacillus sakei*	SHU1387
*Weissella viridescens*	SHU6811
*Lactobacillus reuteri*	SHU1872
*Pediococcus dextrinicus*	SHU1593
*Enterococcus faecium*	SHU1563
*Lactobacillus plantarum*	SHU3455
*Lactobacillus rhamnosus*	SHU1904
*Lactobacillus casei subsp.casei*	SHU2412
*Lactobacillus dextrinicus*	SHU68

To prepare the subcultures, MRS was inoculated with a colony from the plates and incubated overnight at 37 °C. The microbial strains were cultured in 250-ml shake flasks containing 100 ml MRS without Tween 80^®^. Each culture broth was inoculated with 1 ml of an overnight subculture and incubated for 48 h at 37 °C and 120 rpm. After 48 h, the cells were harvested by centrifugation (10,000 × g, 5 min, 10 °C), washed twice in demineralized water, and resuspended in 20 ml phosphate-buffered saline (PBS: 10 mM KH_2_PO_4_/K_2_HPO_4_ and 150 mM NaCl with the adjusted pH of 7.0). In order to release BSs, the cell-suspension was left at room temperature for 8 h in a roller mixer with gentle stirring (60 rpm). Subsequently, the cells were removed by centrifugation (10,000 × g, 5 min, 10 °C) and the remaining supernatant liquid was assayed for the presence of cell-free BS^13,16^.

### Screening of Bacteria for BS Production

#### Hemolytic Essay

A number of blood agar plates containing 5% (v/v) sterile blood were prepared. Then 20 μl of each microbial suspension was added into the 4-mm-diameter wells made in the blood agar. The plates were kept in a refrigerator for 0.5 h to diffuse the spent broth and then were incubated at 37 °C for 24 h. Hemolytic activity was detected as the presence of a clear zone around the wells.

#### Oil-Spreading Method

The oil-spreading method was implemented in triplicates by addition of 50 ml of distilled water to Petri dishes followed by adding 100 μl of vegetable oil to the surface of the water. Afterwards, 10 μl of the cell-free BS solution was dropped on the oil surface. The creation of clear zone on the oil surface was visualized under visible light and measured after 30 s^17^.

#### Drop-Collapse Method

First, 35 μl of the cell-free BS solution was pipetted as a drop onto parafilm, and then the spreading of the drop on the parafilm surface was checked after 15 minutes. Those solutions that gave collapsed drops were scored as positive, indicating BS generation^18,19^.

#### Tensiometry

The ST assays were measured with a tensiometer (Nanometric, Contact angle-101, Iran) working based on the principles of Wilhelmy plate method^20^. The accuracy of the ST measurements was verified using pure water (72.80 ± 0.10 mN/m) before each reading. All measurements were repeated three times and the relevant average values were reported. The surface activity of the produced BS was also expressed as a percentage of the reduction in ST (%STR), calculated as follows:

$$ \% STR=\frac{{\gamma }_{m}-{\gamma }_{c}}{{\gamma }_{m}}\times 100$$

where *γ*_*m*_ and *γ*_*c*_ are respectively the ST values of the control and the BS solutions.

#### Emulsification Assays

The capability of BSs to emulsify n-hexadecane was determined by emulsification assay. For this purpose, equal volumes of the hydrocarbons and aqueous BS solution were mixed with a vortex for 2 min and left to stand for 24 h. The %E_24_ was assessed as a percentage of the height of the emulsified layer (mm) divided by the total height of the liquid column (mm)^21^.

#### Control Solutions

PBS was utilized as a negative control in the screening experiments. Cell-free MRS without Tween 80^®^ was employed as a negative control in the hemolytic activity. Also, 10 g/l Sodium dodecyl sulfate solution (SDS) was used as a positive control in all screening methods^22^.

### BS Production by *P. dextrinicus* SHU1593 and Isolation

MRS-Lac (a medium for cultivation of *Lactobacillus* species where glucose is replaced by lactose) without Tween 80^®^ was used as the control medium for BS production. In addition, molasses and date syrup were employed as low-cost materials to study the production of BS by *P. dextrinicus* SHU1593 strain.

Date syrup was extracted from low-quality and stone-free date fruits according to the procedure described in our previous study^23^. The pH and the °Brix (total soluble solid) of the date syrup and molasses solutions were adjusted to 6.7 and 10, respectively. All media were sterilized by autoclaving at 121 °C for 15 min. For removing the sludges, the solutions were centrifuged (SORVALL, RC-5; USA) at 5,000 × g for 15 min under sterile conditions. The total sugar, the total soluble solids, the pH, as well as the nitrogen, potassium, sodium, magnesium, calcium, zinc, iron, copper, and manganese contents, were quantitatively determined according to the AOAC methods^24^.

In order to produce BS using MRS-Lac, date syrup and molasses media, three batch fermentation processes were implemented. Batch fermentations were put into practice in a 5-liter-capacity fermentor (Infors HT, Minifors, Switzerland) containing 2.5 l production medium inoculated with 1% (v/v) of an overnight subculture and incubated for 48 h at 37 °C. During the fermentation processes, pH was maintained at 6.7 by automatic addition of 2.5 N sodium hydroxide solution, and the agitation speed was set to 120 rpm.

After 48 h, the cells were harvested by centrifugation (10,000 × g, 5 min, 10 °C), washed twice in demineralized water, and resuspended in 500 ml of PBS (pH adjusted to 7.0). The cell-suspensions were left at room temperature for 8 h in a roller mixer with gentle stirring (60 rpm). Afterwards, the microbial cells were pelleted by centrifugation, and the supernatant was filtered through a 0.22-mm filter (Millipore). The solutions containing BSs were dialyzed against demineralized water at 4 °C in a Cellu-Sep© membrane (molecular weight cut-off 6000–8000 Dalton; Membrane Filtration Products, Inc., USA) for 24 h. The aqueous suspension of BS was acidified with 1 M HCl (pH 2.0) and kept at 4 °C for 4 h. The precipitated BS was collected by centrifugation (10,000 × g, 15 min, 4 °C). The precipitate was washed twice with acidic water (pH 2.0) and finally resuspended in distilled water, and then freeze-dried and stored at −20 °C.

### Estimation of Biomass and BS Concentration

Bacterial growth was inspected by measuring the broth optical density at a wavelength of 600 nm. A conventional method of cell dry weight measurement was employed for biomass analysis. A 10-ml sample was poured into pre-weighed tubes and centrifuged at 8000 × g for 10 min. Finally, the cell pellet was dried in an oven at 105 °C for 24 h, and its dry weight was measured subsequently.

The BS concentrations (g/l) were determined employing a calibration curve (ST (mN/m) = −8.65 × concentration (g/l) + 77, r^2^ = 0.973) calculated for surfactin, a commercial BS, below the CMC value with known ST. Within this BS concentration range, the reduction of ST is linear, and it is possible to establish a relationship between the BS concentration and the ST. Surfactin can be utilized as a standard since it is one of the best studied BSs (it lowers the ST of water to 27mN/m at 5.0 × 10^−4^ M) and exhibits proteinaceous characteristics as the BSs produced in this study; therefore, it provides a proper procedure for estimating the BS concentration^25^.

### Examination of the Physical Characteristics of BS

To determine the %E_24_ of the BS produced by *P. dextrinicus* SHU1593, 2 ml of n-hexadecane was added to the same volume of the BS solution, mixed with a vortex for 2 min, and left to stand for 24 h. Then, the %E_24_ was measured as described earlier. Also, the %E_24_ of the produced BS was examined against different substrates like kerosene, soybean oil, sunflower oil, fish oil, olive oil, and hexane.

The CMC value of the BS solution was defined by plotting the broth ST as a function of the logarithm of BS concentration. It was found at the point of the intersection between the two lines best fitted to the pre- and post-CMC data^11^. The BS solutions with concentrations ranging from 0.001 to 10 mg/ml were prepared in PBS (pH 7.0), and the ST of each sample was determined based on the principles of Wilhelmy plate method as described above.

The contact angle (CA) values were assayed by pipetting of 100 μl of the cell-free BS solution as a drop onto parafilm or polystyrene surfaces. A digital camera was positioned forming an angle of 90° with the samples to take macro photos. Image J 1.44 software was used to measure the corresponding CA values.

### Antimicrobial and Anti-adhesive Assays

The antimicrobial activity of the BS against *Escherichia coli*, *Pseudomonas aeruginosa*, *Staphylococcus aureus*, *Bacillus cereus*, *Enterobacter aerogenes* and *Salmonella typhimurium* was determined using agar well diffusion method. Briefly, 60 μl of aqueous solutions of the BS was added into the 4-mm-diameter wells made in the pour plated nutrient broth. The plates were kept in a refrigerator for 0.5 h and then incubated at 37 °C for 16 h. Antimicrobial activity was recognized as the appearance of a clear zone around the wells^14^.

The anti-adhesive activity of the isolated BS ranging from 4 to 25 mg/ml against several microbial strains (the same microorganisms that were employed in the antimicrobial assay) was determined according to the method described by Heinemann *et al*.^26^.

### Stability Studies

To elucidate the pH stability of BS, the freeze-dried BS was dissolved at a concentration of 2.7 mg/ml (the value of CMC) in PBS at different pH values (2.0–12.0), and the ST of each sample was determined by the Wilhelmy plate method at room temperature (25 °C). Furthermore, the stability of the BS to high temperatures was determined. For this purpose, the BS solution was incubated at 121 °C for 15 min and left to get cooled at room temperature, and the ST was measured as described above. All the experiments were carried out in triplicate^27^.

### Structural Characterization of BS

The BS protein content was determined by the method of Bradford^28^ applying Coomassie brilliant blue with bovine serum albumin as a standard. Carbohydrate content was estimated by the phenol-sulfuric acid procedure of Dubois^29^ using D-glucose as a standard, and lipid content was determined by the method of Folch *et al*.^30^. The fatty acids of the BS were converted to their methyl esters (FAMEs), then analyzed in a gas chromatograph (Thermo Finnigan, USA) equipped with a split/splitless injector, a fused silica CP-Sil 88 capillary column (length 100 m × 0.25 mm internal diameter with 0.25 μm film thickness) and a flame ionization detector (FID). The injector temperature of 250 °C was applied for the injection of the 1 μl samples. The column temperature was programmed to be between 140 and 240 °C at a rate of 3.2 °C/min. Nitrogen gas was used as the carrier gas. The FID temperature was kept at 260 °C during the analysis. The fatty acids were identified by comparing the retention times of the methyl esters of the samples with the standard.

Also, the Fourier transforms infrared (FTIR) spectra of the lyophilized samples of the BSs produced by *P. dextrinicus* SHU1593 were taken in the wavelength range of 400–4000 cm^−1^ with a scan speed of 2 mm/s on an FTIR system (Perkin-Elmer Spectrum RX I, USA).

### Statistical Analysis

The mean values of the samples were analyzed statistically via SPSS software (version 22.0). They were compared using one-way ANOVA and Duncan test to reveal any significant differences among the parameters and the variables. Also, the paired t-tests were applied to compare the mean values of CA. They were compared at a significant level of 5%.

## Results and Discussion

### Screening and Selection of BS-Producing Bacteria

As described earlier, eighteen LAB strains were screened using qualitative and quantitative methods regarding BS production. The results of the qualitative assays (See Table 2) revealed that six strains (SHU6811, SHU1593, SHU1563, SHU3455, SHU1904 and SHU68) have the ability to cause complete hemolysis (*β*-hemolysis) in the blood agar culture media. In addition, five strains (SHU6343, SHU1593, SHU3455, SHU1904 and SHU68) were detected to be positive based on the oil-spreading and drop-collapse assays. Also, two strains (SHU6811 and SHU1965) showed positive results only in the drop-collapse experiments. The results of the quantitative assays (See Table 2) revealed that the %E_24_ values of the BS produced by strains SHU1593 and SHU68 were maximal, and the statistical analysis demonstrated that the differences between these values compared with others are significant. Moreover, in the other groups, the %E_24_ values of BS produced by strains SHU3455 and SHU1904 were significantly higher than those of other strains. In the ST experiments, three strains were capable of increasing the percentage of STR to over %35 (strains SHU3455, SHU1593, and SHU1904) and the highest level was obtained to be approximately %40. It was concluded from the various screening assays that the strains of *P. dextrinicus* SHU1593, *L. plantarum* SHU3455 and *L. rhamnosus* SHU1904 were the most efficient BS-producing LAB, which have the ability to produce large amounts of BSs. The strain *P. dextrinicus* SHU1593 was selected for our future studies.

**Table 2:** Results of qualitative and quantitative screening assays for BS production.

Strain	Hemolytic test	Oil spreading method	Drop-collapse method	%E_24, hexadecane_*	ST (mN/m)**	%STR
SHU1388	γ	—	—	0	62.01 ± 0.01	14.82^k^ ± 0.02
SHU1365	γ	—	—	0	63.47 ± 0.44	12.81^l^ ± 0.60
SHU1400	γ	—	—	0	62.12 ± 0.19	14.66^k^ ± 0.26
SHU1391	α	—	—	0	57.51 ± 0.49	21.00^h^ ± 0.67
SHU1394	α	—	—	5.88^g^ ± 0.85	54.04 ± 1.22	25.76^f^ ± 1.67
SHU1396	γ	—	—	4.18^g^ ± 0.32	54.26 ± 1.53	25.45^f^ ± 2.11
SHU1965	α	—	+	17.26^e^ ± 0.42	50.83 ± 0.15	30.17^de^ ± 0.20
SHU6343	γ	+	+	21.13^d^ ± 0.24	50.17 ± 0.15	31.08^d^ ± 0.21
SHU1368	α	—	—	1.77^h^ ± 0.61	57.13 ± 0.15	21.52^h^ ± 0.20
SHU1387	α	—	—	0	62.38 ± 0.32	14.30^k^ ± 0.44
SHU6811	β	—	+	9.50^f^ ± 1.00	50.70 ± 0.29	30.35^de^ ± 0.40
SHU1872	α	—	—	0	60.64 ± 0.19	16.69^j^ ± 0.26
SHU1593	β	+	+	43.04^a^ ± 2.39	44.98 ± 0.26	38.20^b^ ± 0.36
SHU1563	β	—	—	0	58.52 ± 0.54	19.61^i^ ± 0.74
SHU3455	β	+	+	38.79^b^ ± 2.90	44.08 ± 0.12	39.44^a^ ± 0.17
SHU1904	β	+	+	26.38^c^ ± 1.47	46.85 ± 0.27	35.64^c^ ± 0.37
SHU2412	α	—	—	1.41^h^ ± 0.61	55.15 ± 0.25	24.23^g^ ± 0.34
SHU68	β	+	+	43.61^a^ ± 3.19	51.51 ± 0.50	29.23^e^ ± 0.68

Values are representatives of mean ± S.D. (n = 3). Values followed by different superscripts in a column are significantly different (P < 0.05). *%E_24, hexadecane/SDS_ = 65%. **ST of PBS was 72.80 ± 0.10 mN/m.

The blood agar lysis method was employed in this study as it has been widely used for screening purposes in BS production solely or with additional assays. Previous studies have demonstrated that almost all BS producers have positive hemolytic activity, whereas not all hemolytic species are BS producers^17^. Therefore, the hemolytic activity must be considered as a preliminary test and an unreliable criterion to detect the presence of any BS molecule in a microbial culture. The oil-spreading method is based on the phenomenon that a drop of BS-containing solution can replace the oil and as a consequence can spread in water. In the drop-collapse method, the drops which include BS, spread or even collapse due to the reduction of the interfacial tension between the liquid drop and the hydrophobic surface^17,31^. In this study, it was shown that the hemolytic activity assay has a relatively obvious relationship with the qualitative oil-spreading and drop-collapse tests. Regarding the emulsification assays, in order to form an emulsion, BSs must significantly lower the oil-water interfacial tension. Furthermore, BSs must rapidly diffuse towards the newly created interface^32^. The potential of a BS is assessed by its ability to reduce the ST value of the productive medium^33^. According to the literature, the measurement of ST seems to be the only reliable method for screening BS-producing strains.

In the current study, we utilized several screening methods to find the most operative BS-producer to be properly employed in food industry sectors. For instance, a number of researchers have notified that the ability of a molecule to form a stable emulsion is not always associated with its ST lowering activity^34^. Hence, the use of a combination of several screening methods could help us to choose a BS which exhibits various characteristics such as high emulsifying and wetting properties. In practice, there exist complexities to evaluate the results of the screening experiments, especially when the number of the deliberated strains is high.

### Effect of the Carbon Source

The production of BS was carried out in a bioreactor by *P. dextrinicus* SHU1593 (the selected strain from the screening assays), using different carbon sources. MRS-Lac, date syrup, and molasses were evaluated as the media for BS biosynthesis as described before. The ingredients of the utilized molasses and date syrup were quantitatively examined (See Table 3), and the carbon/nitrogen (C/N) ratio of the date syrup and molasses media were calculated to be 46.40 and 26.50, respectively. It was found that the molasses was more nutritious than the date syrup due to the high levels of suitable elements, especially calcium and iron contents. Therefore, it is expected that they will be adequate substrates for LAB growth since LAB are fastidious and require complex growth factors. Sugar beet molasses is a worthwhile source of growth substances such as pantothenic acid, inositol, trace elements and to a lesser extent, biotin. Consequently, it is broadly used as a low-cost substrate in fermentative processes. However, some contaminants such as biocides and heavy metals have been identified in sugar beet molasses, but most of them are at concentration levels below than those significantly affecting the process restraint^35^. The dates and the date syrup are used for human consumption, in bakery and ice-cream products, and for the synthesis of ethanol, caramel color, vinegar, and single cell protein. Because of high concentrations of sugars present in the date syrup, it is important to develop new uses of these sugars, especially for fermentation purposes^36^.

**Table 3:** Composition of the molasses and date syrup used as substrate for microbial growth and BS production.

Parameter	Molasses	Date syrup
°Brix	10	10
pH	5.93	4.35
Total sugar (%)	6.8	8.3
Protein (%)	0.83	0.52
Calcium (mg/l)	708.3	79.03
Zinc (mg/l)	1.105	0.359
Iron (mg/l)	7.290	0.168
Copper (mg/l)	0.045	0.107
Manganese (mg/l)	0.312	0.136
Magnesium (mg/l)	217.8	196
Sodium (mg/l)	81.1	46
Potassium (mg/l)	72.5	45.5

**Figure 1:**
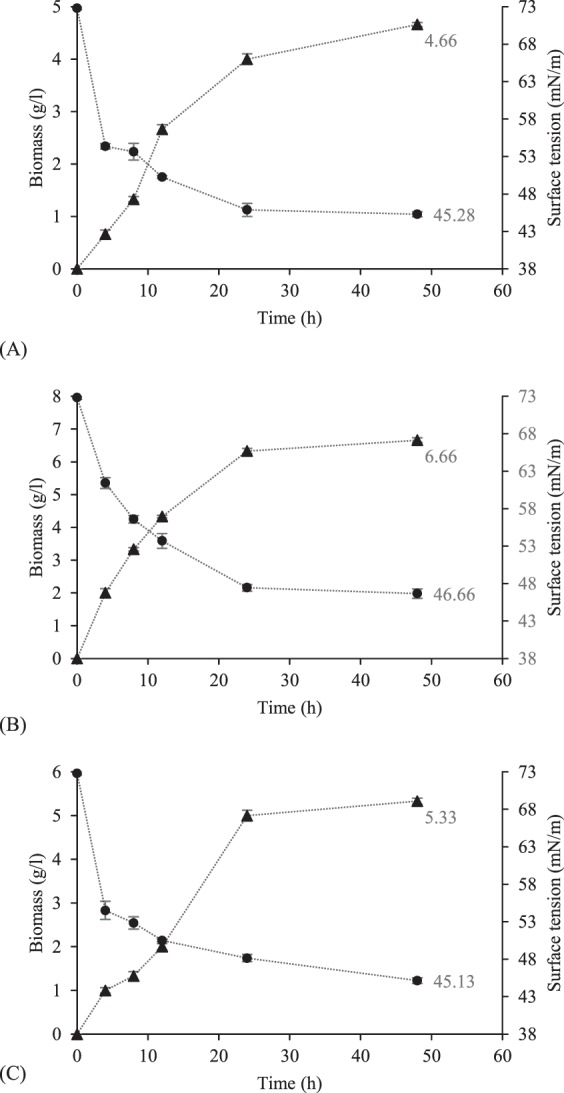
ST variation (mN/m) of BS solutions and biomass concentrations (g/l) obtained from fermentations carried out in the bioreactor using MRS-Lac (**A**) date syrup (**B**) and molasses (**C**) media. Results represent the average of three independent experiments. Biomass (..▲..); ST (..•..).

At the end of the fermentation processes, the obtained biomass concentrations were 4.66 g/l, 6.66 g/l and 5.33 g/l for MRS-Lac, date syrup and molasses media, respectively, while the ST values of all three BS solutions reduced from 72.80 mN/m to about 45 mN/m (the measured ST values were below the CMC value) (See Fig. 1). It was observed that the various employed carbon sources resulted in different biomass concentrations. The date syrup and molasses as carbon sources instead of the conventional medium, MRS-Lac, stimulated the cells for more proliferation; however, they did not induce the cells to produce more BS. Hence, assuming that the BSs produced in various media are structurally similar, the method of measurement of biomass concentrations in different culture media for screening the production of BS, is not necessarily adequate. The common sugars present in the MRS-Lac, date syrup, and molasses media are respectively lactose, invert sugar and sucrose^35,37^. LAB which ferment sugars are also capable of accumulating various products via different pathways, e.g. flavors such as diacetyl and acetoin, bacteriocins or BSs. The different carbon sources contribute to varying amounts of by-products^38,39^. It can be hypothesized that the utilization of lactose or sucrose as the main carbon sources instead of invert sugar induces the cells to employ other metabolic pathways, and accordingly produces different amounts of BS. Moreover, it can be speculated that the higher ratio of C/N (date syrup medium) leads to a higher generation of biomass and restricts the biosynthesis of BS. It is obviously seen in the literature that the C/N ratio plays an indisputable role in the production of rhamnolipids^40^ and other BSs^41^. Also, it has been found that the molasses was more nutritious than the date syrup due to the high levels of suitable elements; hence, the presence of these ingredients might affect the synthesis of BSs. The trace elements serve as a co-factor for the enzymes involved in the BSs biosynthesis. Onur^42^ reported that the supplementation of low-cost media with Fe^2+^, Mn^2+^, and Mg^2+^, could be advantageous for BS synthesis.

In this study, a direct relationship was observed between BS production (shown by a decrease in the ST value) and cell growth during all three fermentation processes. Thus, BS biosynthesis was found to be growth-associated which is in accordance with earlier reports^43,44^. Also, the produced BS reached a constant level 24 hours after the startup of the fermentation processes. Production of BSs in a short time (24 hours) is considered to be beneficial from various aspects: saving energy costs, reducing depreciation costs of the required devices, and synthesis of more products in a monthly work schedule.

In this part of the study, we focused on the potential use of alternative fermentation media for BS production. The final BS concentrations in all three producing media were found to be approximately 0.7 g/l. Although the amounts of the produced BS per liter of culture medium were approximately equivalent in all three media, the results are economically different. On average, 10 liters of the substrate (°Brix 10) can be prepared from each kilogram of the initial molasses or date fruits. The cost for preparing one liter of the utilized waste media, date syrup or molasses, is just 0.05% of the cost for providing one liter of the synthetic culture medium. Hence, utilizing waste media instead of synthetic MRS-Lac, contributes to 99% saving in costs of medium preparation.

The synthesis of LAB-derived BSs from various agro-industrial wastes is an encouraging route to attain low-cost bioprocesses. Gudiña *et al*.^27^ reported that cheese whey, a relatively low-priced and easily available resource, can be used for the BS production by *Lactobacillus agilis CCUG31450*. Supplemented cheese whey and molasses media were successfully utilized for the BS production by the probiotic strains, *Lactococcus lactis* 53 and *Streptococcus thermophilus* A^25^. In another study, monomeric hemicellulosic sugars obtained from diluted acid hydrolysis of distilled grape marc were used to be efficiently converted to BSs by *Lactobacillus pentosus* after nutrient supplementation^45^. Moreover, hemicellulosic sugars obtained from agricultural residues such as trimming vine shoots or detoxified *Eucalyptus globulus* wood hydrolyzates were successfully used as carbon sources for the production of BSs using LAB^46^.

To our knowledge, very few studies have been conducted on the use of date syrup as a substrate for the economic synthesis of microbial products. In our recent study, we performed a fed-batch fermentation to develop an enhanced BS production with low-quality date syrup by *Lactobacillus rhamnosus*^23^. Al-Bahry *et al*.^47^ reported BS production with date molasses by *Bacillus subtilis* B20 and investigated its possible application in oil recovery. Another study examined production of BS on crude date syrup under saline conditions by entrapped cells of *Natrialba* sp. strain E21, which is an extremely halophilic bacterium isolated from a solar saltern^48^.

### Physical Characteristics of BS

**Figure 2:**
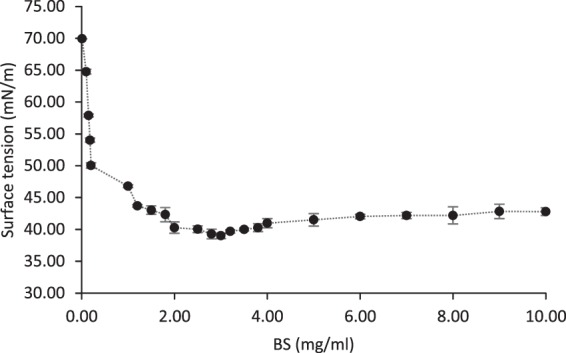
Progressive decrease in ST with increase in BS concentration up to 2.7 mg/ml.

**Figure 3:**
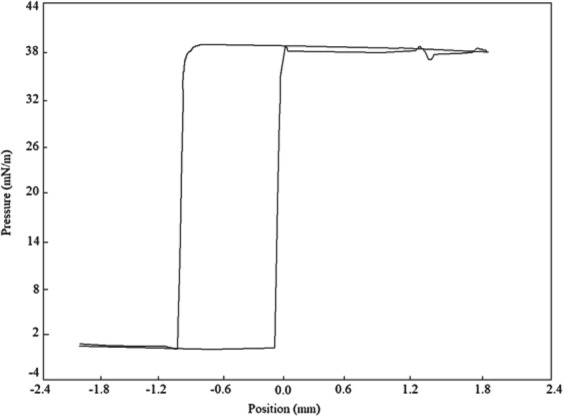
ST value of 39.01 calculated at distance between −2 and 2 of the BS solution obtained from *P. dextrinicus* SHU1593. The surface of the BS solution corresponds to zero level.

The freeze-dried BS produced by *P. dextrinicus* SHU1593 was dissolved in PBS (pH 7.0) at different concentrations ranging from 0.001 to 10 mg/ml (See Fig. 2). A gradual decline in ST was observed as a result of raising the quantity of BS concentration. For BS concentrations higher than 2.7 mg/ml, the ST turned into a relatively constant state; hence, this value is considered as the CMC for this BS. The BS showed a significant reduction in ST value of PBS from 72.80 ± 0.10 to 39.01 ± 0.32 mN/m. As it can be seen in Fig. 3, as soon as the platinum plate came into contact with the surface of BS solution (the zero point), the value of ST abruptly reached 39.01 ± 0.32 and then precipitously decreased to zero after exiting the platinum plate from the BS solution. Since a potent surface-active agent has a low CMC value, a very low concentration of it is required to decrease the ST value^49^. At low concentrations, amphiphilic molecules can be arrayed on the surface of the aqueous phase, and therefore their polar moieties interact with the soluble phase, and their nonpolar moieties are placed above the surface (in the air/nonpolar liquid)^50^. When the surface is saturated with these molecules, additional molecules come together in structures called micelle. In fact, due to fragile hydrophobic and van der Waals contacts, they combine and entangle hydrophobic parts and emulsify in water^51^. Accordingly, when the BS produced by *P. dextrinicus* SHU1593 is dissolved in PBS (pH 7.0), at concentrations of higher than 2.7 mg/ml, some molecules come together and form micelle structures. The minimum ST (39.01 ± 0.32 mN/m) and the CMC (2.7 mg/ml) obtained herein were found to be similar to the values previously obtained for other LAB-derived BSs^5,9,10^.

**Figure 4:**
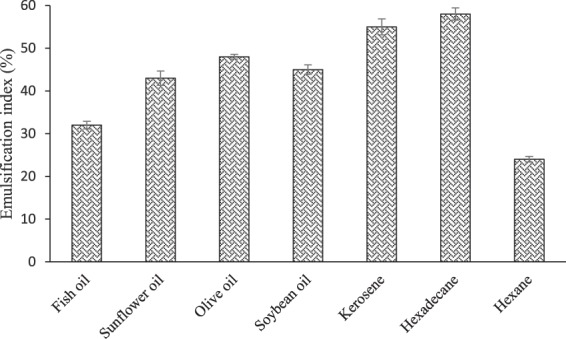
%E_24_ of the BS for various substrates. Results are expressed as mean ± standard deviations of values from triplicate experiments.

Examined in the present study were also the capabilities of BS to emulsify n-hexadecane, kerosene, soybean oil, sunflower oil, fish oil, olive oil, and hexane (See Fig. 4). The maximum %E_24_ (58 ± 1.40) was found for BS/n-hexadecane emulsion. The results revealed that kerosene and n-hexadecane were significantly (more than 50%) emulsified, while the lowest emulsification indices were obtained for hexane and fish oil. The %E_24_ against certain vegetable oils like soybean oil, sunflower oil, and olive oil which are widely utilized in food formulations were measured to be 45 ± 1.1, 43 ± 1.65 and 48 ± 0.55, respectively. In a similar study, Madhu and Prapulla^14^ reported that the BS produced by *L. plantarum* CFR 2194 is able to emulsify coconut oil (37.9%) and sunflower oil (19.43%).

The CA values of the drops containing the BS produced by *P. dextrinicus* SHU1593, and pipetted onto the surfaces of polystyrene and parafilm were reduced from 98.86° ± 0.58 and 103.93° ± 0.52 (for the control samples) to 72.92° ± 0.65 and 73.24° ± 1.06, respectively (The values for the CAs were significantly different, p-value < 0.05). When a liquid comes into contact with a solid surface, it forms a CA with the surface. The CA relies on the attributes of the solution and the solid. If a wetting agent is added to the water to reduce its ST, and the water drop comes into contact with a hydrophobic surface, the drop makes a smaller CA, and therefore a greater portion of the surface gets wet. The spreading of the drop containing the BS is due to the reduction of the interfacial tensions between the drop and the hydrophobic surface, and therefore, the produced BS would be effective as a partial wetting agent.

### Antimicrobial and Anti-Adhesive Effects of BS

In this study, we observed that an increase in the BS concentration led to improvement of its antimicrobial effects (Table 4). The highest antimicrobial effects of BS were observed against *E. aerogenes* and *E. coli*, respectively. The BS at a concentration of 25 mg/ml exhibited a complete antimicrobial effect against *E. coli*, *E. aerogenes*, and *P. aeruginosa*. However, it did not exhibit any significant impacts on *B. cereus* and *S. aureus*. Therefore, the results demonstrated the antimicrobial potential of BSs generated by *P. dextrinicus* SHU1593, mainly in high concentrations.

**Table 4:** Antimicrobial and anti-adhesive activities of the BS derived from *P. dextrinicus* SHU1593.

Microorganism	Antimicrobial assays		Anti-adhesive assays	
	4 mg/ml	25 mg/ml	4 mg/ml	25 mg/ml
*B. cereus*	−	−	22.53 ± 0.20	70.50 ± 0.38
*E. coli*	+	++	4.00 ± 0.62	44.25 ± 0.18
*E. aerogenes*	++	++	5.22 ± 0.43	29.87 ± 0.25
*P. aeruginosa*	−	++	17.12 ± 0.10	61.84 ± 0.27
*S. typhimurium*	−	+	12.88 ± 0.26	58.69 ± 0.10
*S. aureus*	−	−	8.59 ± 0.17	53.18 ± 0.33

“ + ” sign shows inhibition of microbial growth, whereas “−” sign means an inability to inhibit the growth of the examined microorganisms. Values of anti-adhesive assays are representatives of mean ± S.D. (n = 3).

BSs have been considerably dealt with in recent years as they form an incipient group of novel antimicrobial compounds. They can be employed as safe and effective therapeutic compounds; thus, they may be appropriate alternatives to conventional antibiotics. The antimicrobial characteristic of BSs is due to the potential of these molecules to self-associate and create pores inside the cell membrane structure^52^. Various LAB-derived BSs have shown antimicrobial attributes. Gudiña *et al*. reported that the BS-derived from *Lactobacillus agilis* CCUG31450 exhibited antimicrobial characteristics at a concentration of 5 mg/ml against *S. aureus*, *P. aeruginosa*, and *S. agalactiae*^27^. Also, it was found in other studies that BSs obtained from *S. thermophilus* A and *L. lactis* 53 exhibited remarkable antimicrobial properties against various pathogens isolated from voice prostheses^13,16^. BSs of both *L. jensenii* and *L. rhamnosus* revealed antimicrobial activities against *A. baumannii*, *E. coli* and *S. aureus* at 25–50 mg/ml^12^.

The isolated BS was found to possess a considerable anti-adhesive activity against all the microorganisms examined (See Table 4). The highest anti-adhesive impacts were obtained for *B. cereus* (70.50%), *P. aeruginosa* (61.84%) and *S. typhimurium* (58.69%) for BS concentration of 25 mg/ml. On the contrary, limited activities were observed for *E. coli* (44.25%) and *E. aerogenes* (29.87%) at the same BS concentration. Prior adsorption of BSs on solid surfaces might establish an effective strategy to decrease microbial adhesion and inhibit colonization of pathogenic microorganisms. LAB-derived BSs have been reported to have remarkable anti-adhesive and biofilm disruption properties^5,12,53^.

### Temperature and pH Stability of the Synthesized BS

**Figure 5:**
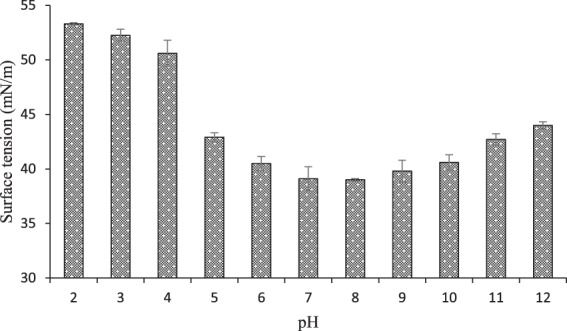
Stability study of BS derived from P. dextrinicus SHU1593 at different pH values. Results are expressed as mean ± standard deviations of values from triplicate experiments.

The applicability of BSs mainly relies on their behavior at different temperatures and pH exposures. The produced BS in the present study was found to be very stable when exposed to high temperatures since no significant statistical difference was observed between the %STR values of the BS before and after a 15-minute incubation at 121 °C. The most favorable surface activities of the synthesized BS were found over a pH range from 6 to 10, although the highest was observed at pH 8 (See Fig. 5). As pH decreases, the ST continues raising up to 53.3 ± 0.1 mN/m at pH 2. In other words, the surface activity of the BS remained relatively more stable at alkaline conditions in comparison to acidic ones. Furthermore, for pH values lower than 6, turbidity appeared in the samples due to the relative precipitation of the BS.

The higher persistence of BSs generated by some LAB under alkaline conditions, as well as their instability under acidity, has also been mentioned and described by other researchers^11,13,16^. The BS lability state at acidic conditions stems from protonation of negatively-charged groups located at the polar terminals of the molecules.

### BS Characterization

Our chemical composition examinations disclosed that the BS generated by *P. dextrinicus* SHU1593 is most likely a lipoprotein with an approximately equal ratio of lipids (~52% (w/w)) and proteins (47% (w/w)). A negligible fraction of carbohydrates (less than 1%) was detected in some of the obtained samples possibly due to the remaining culture media co-precipitated with the BS during its extraction process. Besides, the chemical analysis revealed that the predominant fatty acids contained in the BS were oleic (60.28%), palmitic (25.80%), stearic (7.43%) and lauric (4.60%) with the retention time values of 28.07, 22.78, 26.88 and 18.61 minutes, respectively. The production of lipoprotein BS employing LAB has not yet been commonly reported. Instead, BSs in different studies were found to be glycolipids^5,13^, glycoproteins^14^ or a multi-component mixture of proteins and polysaccharides associated with phosphate groups^10^. Vecino *et al*.^54^ examined the BS produced by *L. pentosus* and reported it as a mixture of carbohydrate, protein, and lipid with a combination ratio of 1:3:6. Their study showed that linoelaidic, palmitic, and stearic acids were the major fatty acid types in the BS generated.

**Figure 6:**
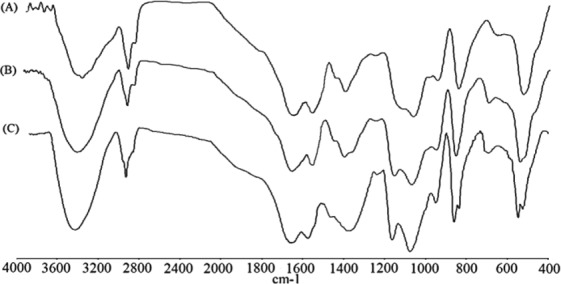
FTIR absorption spectra of BSs produced by strain SHU1593 in MRS-Lac (**A**), date syrup (**B**) and molasses (**C**) media.

The molecular characterizations of the BSs produced by *P. dextrinicus* SHU1593 in the MRS-Lac, date syrup, and molasses media were also carried out using FTIR spectroscopy. The FTIR spectra (Fig. 6) indicated that all of the three BSs produced are significantly similar in nature. The detected peaks were comparatively similar to the IR spectrum of lipopeptide BSs generated by *Bacillus* species^7^. The signal at the band of 1075 cm^−1^ suggested the presence of amide moieties of proteins^55^. The most prominent bands located at the range of 3000–3600 cm^−1^ and 1640–1700 cm^−1^, as well as 1500–1620 cm^−1^, correspond to NH group, C=O stretching in proteins (AmI band) and NH bending in proteins (AmII band), respectively, which reveal the presence of proteins in the BS samples. Absorption in 1500–1620 cm^−1^ is not normally discerned in the FTIR spectra of glycolipid BSs^56^. The peaks in the FTIR spectra at 2927, 2929 and 2924 cm^−1^ correspond to C–H bands (CH_2_–CH_3_ stretching). The small peaks in the region of 1370–1470 cm^−1^ are the result of deformation and bending vibrations of –C–CH_2_ and –C–CH_3_ groups in aliphatic chains. Furthermore, peaks at 1127 and 1250 cm^−1^ are probably due to C–O–C vibrations in esters. The spectra obtained from the BSs produced by *P. dextrinicus* SHU1593 in the MRS-Lac, date syrup, and molasses media suggest that the BSs are composed of lipid and protein.

The FTIR spectroscopy analysis of the BSs produced in the three examined media indicated that the obtained BSs are not structurally different, although the media are dissimilar in character and composition. In other words, the BSs produced in the three media were not significantly influenced by changing the substrates employed.

## Conclusions

In the present study, we focused on producing and characterizing a type of LAB-derived BS (using *P. dextrinicus* SHU1593) and its potential applications in pharmaceutical and food industries due to the preference of employing nonpathogenic organisms in bioprocesses. The results indicated the potential of developing low-cost strategies aimed at the production of efficient LAB-derived BSs which are structurally and quantitatively similar to the ones obtained from conventional media. The results also indicated that while the nature (lipoprotein) and amounts (~0.7 g/l) of the BSs produced in the conventional and alternative media were similar, the biomass formation rate was different in those media. It is worth noting that although the BS production yield by *P. dextrinicus* SHU1593 was low, this work can be considered as an opening project and complementary studies should be performed regarding genetic manipulations and metabolic engineering as well as development of efficient operating strategies for enhancement of the BS productivity, yield and titer. Moreover, given the physical and functional characterization of the BS produced in the present study, it can be introduced as a promising candidate to be employed in plenty of areas in pharmaceutical and food industries.
