# Author Correction: Biosurfactant Production by Lactic Acid Bacterium *Pediococcus dextrinicus* SHU1593 Grown on Different Carbon Sources: Strain Screening Followed by Product Characterization

**DOI:** 10.1038/s41598-020-58139-8

**Published:** 2020-01-21

**Authors:** Abouzar Ghasemi, Marzieh Moosavi-Nasab, Payam Setoodeh, Gholamreza Mesbahi, Gholamhossein Yousefi

**Affiliations:** 1 Department of Food Science and Technology, School of Agriculture, Shiraz University, Shiraz, Iran; 2 Seafood Processing Research Group, School of Agriculture, Shiraz University, Shiraz, Iran; 3 Department of Chemical Engineering, School of Chemical and Petroleum Engineering, Shiraz University, Shiraz, Iran; 4 Department of Pharmaceutics, School of Pharmacy, Shiraz University of Medical Sciences, Shiraz, Iran

Correction to: *Scientific Reports* 10.1038/s41598-019-41589-0, published online 27 March 2019

In this Article, the legend of Figure 5 is incorrect:

“Stability study of BS derived from P. dextrinicus SHU1593 at different pH values. Results are expressed as mean ± standard deviations of values from triplicate experiments.”

should read:

“Stability study of BS derived from *P. dextrinicus* SHU1593 at different pH values. Results are expressed as mean ±  standard deviations of values from triplicate experiments.”

Additionally, this Article contains a typographical error in the Author Contributions section.

“M.M.N.; Advisers: P.S., G.M., G.Y.; Writing draft: A.G.; Writing review, editing and revision: A.G., M.M.N., P.S.; Funding acquisition: M.M.N.; Software: A.G., M.M.N., P.S.; Data analysis: A.G., M.M.N., P.S.”

should read:

“Supervisor: M.M.N.; Advisers: P.S., G.M., G.Y.; Writing draft: A.G.; Writing review, editing and revision: A.G., M.M.N., P.S.; Funding acquisition: M.M.N.; Software: A.G., M.M.N., P.S.; Data analysis: A.G., M.M.N., P.S.”

